# Squark and gluino production cross sections in $pp$ collisions at $\sqrt{s} = 13, 14, 33$ and $100$ TeV

**DOI:** 10.1140/epjc/s10052-014-3174-y

**Published:** 2014-12-04

**Authors:** Christoph Borschensky, Michael Krämer, Anna Kulesza, Michelangelo Mangano, Sanjay Padhi, Tilman Plehn, Xavier Portell

**Affiliations:** 1 Institut für Theoretische Physik, Westfälische Wilhelms-Universität Münster, 48149 Münster, Germany; 2 Institute for Theoretical Particle Physics and Cosmology, RWTH Aachen University, 52056 Aachen, Germany; 3 European Organization for Nuclear Research, CERN, Meyrin, Switzerland; 4 University of California, San Diego, USA; 5 Institut für Theoretische Physik, Universität Heidelberg, Heidelberg, Germany

## Abstract

We present state-of-the-art cross section predictions for the production of supersymmetric squarks and gluinos at the upcoming LHC run with a centre-of-mass energy of $\sqrt{s} = 13$ and $14$ TeV, and at potential future $pp$ colliders operating at $\sqrt{s} = 33$ and $100$ TeV. The results are based on calculations which include the resummation of soft-gluon emission at next-to-leading logarithmic accuracy, matched to next-to-leading order supersymmetric QCD corrections. Furthermore, we provide an estimate of the theoretical uncertainty due to the variation of the renormalisation and factorisation scales and the parton distribution functions.

## Introduction

The search for supersymmetry (SUSY) is a central activity of the LHCphysics programme. To date, a variety of experimental searches have been performed at the collision energies of $7$ and $8$ TeV and a broad range of possible final states has been examined [1, 2].

In the framework of the Minimal Supersymmetric extension of the Standard Model (MSSM) with R-parity conservation, SUSY particles are produced in pairs. At the LHC, the most copiously produced SUSY particles are expected to be the strongly interacting partners of quarks, the squarks ($\tilde{q}$), and the partners of gluons, the gluinos ($\tilde{g}$). The dominant squark and gluino pair-production processes are

1 $$\begin{aligned} pp \rightarrow \tilde{q}\tilde{q}, \tilde{q}\tilde{q}^*, \tilde{q}\tilde{g}, \tilde{g}\tilde{g}+ X \,, \end{aligned}$$

together with the charge conjugated processes. In Eq. (1) the chiralities of the squarks, $\tilde{q}=(\tilde{q}_L,\tilde{q}_R)$, are suppressed, and we focus on the production of the partners of the $(u,d,c,s,b)$ quarks which we assume to be mass degenerate. The production of the SUSY partners of top quarks, the stops $(\tilde{t})$, and, when appropriate, the partners of bottom quarks, the sbottoms $(\tilde{b})$, has to be considered separately due to parton distribution function (PDF) effects and potentially large mixing affecting the mass splittings. In this case, we explicitly specify the different mass states in the pair-production processes,

2 $$\begin{aligned} pp \rightarrow \tilde{t}_i \tilde{t}^*_i, \tilde{b}_i \tilde{b}^*_i + X \qquad \qquad i=1,2\,, \end{aligned}$$

where $i=1,2$ corresponds to the lighter and heavier states, respectively.

Given the importance of SUSY searches at the LHC, accurate knowledge of theoretical predictions for the cross sections is required. Starting from mid-2011, ATLAS and CMS analyses have been based on resummed results at the next-to-leading logarithmic (NLL) accuracy matched to next-to-leading order (NLO) predictions, referred to as NLO + NLL in the rest of this paper.

With minimal assumptions on SUSY productions and decays, the interpretation of the current LHCdata with $\sqrt{s} = 7$ and $8$ TeV leads to the mass bound for the gluino and light squarks as $m_{\tilde{g}} = m_{\tilde{q}} > 1.7$ TeV, or $m_{\tilde{g}} > 1.4$ TeV with a decoupled squark sector, and $m_{\tilde{q}} > 850$ GeV with the other decoupled particles, based on the ATLAS/CMS studies. Pair productions of SUSY third-generation lighter states also have a mass bound above $m_{\tilde{t}, \tilde{b}} > 750$ GeV. This paper provides a reference for the evaluation of SUSY squark and gluino production cross sections and their theoretical uncertainties for the extended range of superpartner masses within the reach of the upcoming LHC runs at $\sqrt{s}=13$ and $14$ TeV, and for future high-energy hadron colliders at $\sqrt{s}=33$ and $100$ TeV. The paper follows [3] in which results for $\sqrt{s}=7$ TeV were presented. The detailed cross section values for the relevant processes and SUSY models considered by the experiments, as well as the results for lower LHC centre-of-mass energies, are collected at the SUSY cross section working group web page [4].

The next section briefly describes the current state-of-the-art higher-order calculations for squark and gluino hadroproduction, followed by the prescription used for the treatment of theoretical uncertainties in Sect. 3. In Sect. 4 the production cross sections are presented, and a summary of the results and of the future prospects is given in Sect. 5.

## Higher-order calculations–NLO + NLL

The dependence of hadron collider observables on the renormalisation and factorisation scales is an artifact of perturbation theory and is generically reduced as higher-order perturbative contributions are included. Assuming that there is no systematic shift of an observable from order to order in perturbation theory, for example due to the appearance of new production channels, the range of rates covered by the scale dependence at a given loop order should include the true prediction of this rate. The scale dependence therefore provides a lower limit on the theory uncertainty of a QCD prediction, which becomes smaller as higher-order SUSY-QCD corrections are included. To estimate the scale uncertainty in this study we vary simultaneously factorisation and renormalisation scales, within a range of 0.5–2 times the reference central scale $\mu$, where $\mu$ is the average of the two sparticle masses in the final state.

The corrections often increase the size of the cross section with respect to the leading-order prediction [5–7] if the renormalisation and factorisation scales are chosen close to the average mass of the produced SUSY particles. As a result, the SUSY-QCD corrections have a substantial impact on the determination of mass exclusion limits and would lead to a significant reduction of uncertainties on SUSY mass or parameter values in the case of discovery; see e.g. [8]. The processes listed in Eqs. (1) and (2) have been known for quite some time at NLO in SUSY-QCD [9–12]. Note that SUSY-QCD corrections can be split into two parts. First, there are QCD corrections induced by gluon or quark radiation and by gluon loops, which follow essentially the same pattern as, for example, top pair production. Second, there are virtual diagrams which involve squark and gluino loops, and which are independent of the real emission corrections. For heavy squarks and gluinos, the virtual SUSY loops are numerically sub-leading, albeit challenging to compute. This is mainly due to a large number of Feynman diagrams with different mass scales contributing to the overall cross section. For stop pair production, where neither light-flavour squarks nor gluinos appear in the tree-level diagrams, the virtual SUSY contributions can easily be decoupled [12]. The only part that requires some attention is the appropriate treatment of the counter term and the running of the strong coupling constant. This decoupling limit is implemented in Prospino2 [13]. For light-flavour squark and gluino production this decoupling would only be consistent if applied to the leading order as well as NLO contributions. This is usually not required, unless we choose specific simplified models.

Given the expected squark flavour structure in the MSSM, most numerical implementations, including Prospino2, make assumptions as regards the squark mass spectrum. The left-handed and right-handed squarks of the five light flavours are assumed to be mass degenerate. Only the two stop masses are kept separate in the NLO computations of light-flavour production rates [9–11]. In the Prospino2 [13] implementation, this degeneracy is not assumed for the leading-order results. However, the approximate NLO rates are computed from the exact leading-order cross sections times the mass-degenerate $K$-factors, i.e. the ratio of NLO and LO cross section for mass-degenerate squarks. For the pair production of third-generation squarks the four light squark flavours are assumed to be mass degenerate, while the third-generation masses are kept separate [12]. This approximation can for example be tested using MadGolem [14], an automatised NLO tool linked to MadGraph4 [15], or other recent NLO calculations that keep all squark masses separate [16–19]. It is also important to point out here that in Prospino2 the pair production of third-generation squarks is available as an individual processes. However, sbottom pairs are included in the implicit sum of light-flavour squarks because there is no perfect separation of bottom and light-flavour decay jets.

When summing the squark and gluino production rates including next-to-leading order corrections it is crucial to avoid double counting of processes. For example, squark-pair production includes $\mathcal {O}(\alpha _{\mathrm {s}}^3)$ processes of the kind $qg \rightarrow \tilde{q}\tilde{q}^*q$. The same final state can be produced in $\tilde{q}\tilde{g}$ production when the on-shell gluino decays into an antisquark and a quark. The Prospino scheme for the separation and subtraction of on-shell divergences from the $\tilde{q}\tilde{q}^*$ process uniquely ensures a consistent and point-by-point separation over the entire phase space; see also [19]. For a finite particle mass this scheme has recently been adopted by MC@NLO [20] for top quark processes. It is automatised as part of MadGolem [14].

A significant part of the NLO QCD corrections can be attributed to the threshold region, where the partonic centre-of-mass energy is close to the kinematic production threshold. In this case the NLO corrections are typically large, with the most significant contributions coming from soft-gluon emission off the coloured particles in the initial and final state. The contributions due to soft-gluon emission can be consistently taken into account to all orders by means of threshold resummation. In this paper, we discuss results where resummation has been performed at next-to-leading logarithmic (NLL) accuracy [21–25].

The step from NLO to NLO + NLL is achieved by calculating the NLL-resummed partonic cross section $\tilde{\sigma }^\mathrm{(NLL)}$ and then matching it to the NLO prediction, in order to retain the available information on other than soft-gluon contributions. The matching procedure takes the following form:

3 $$\begin{aligned}&\sigma ^\mathrm{(NLO~+~NLL)}_{p p \rightarrow kl}\left( \rho , \{m^2\},\mu ^2\right) = \sigma ^\mathrm{(NLO)}_{p p \rightarrow kl}\left( \rho , \{m^2\},\mu ^2\right) \nonumber \\&\quad \!+\!\, \frac{1}{2 \pi i} \sum _{i,j=q,\bar{q},g}\, \int _\mathrm {CT}\,\mathrm{d}N\,\rho ^{-N}\, \tilde{f}_{i/p}(N\!+\!1,\mu ^2)\,\tilde{f}_{j/p}(N\!+\!1,\mu ^2) \nonumber \\&\quad \times \, \left[ \tilde{\sigma }^\mathrm{(NLL)}_{ij\rightarrow kl}\left( N,\{m^2\},\mu ^2\right) \,-\, \tilde{\sigma }^\mathrm{(NLL)}_{ij\rightarrow kl}\left( N,\{m^2\},\mu ^2\right) {\left. \right| }_{\scriptscriptstyle ({\mathrm {NLO}})}\, \right] ,\nonumber \\ \end{aligned}$$

where the last term in the square brackets denotes the NLL-resummed expression expanded to NLO. The symbol $\{m^2\}$ stands for all masses entering the calculations and $\mu$ is the common factorisation and renormalisation scale. The resummation is performed in the Mellin-moment $N$ space, with all Mellin-transformed quantities indicated by a tilde. In particular, the Mellin moments of the partonic cross sections are defined as

4 $$\begin{aligned} \tilde{\sigma }_{i j \rightarrow kl}\left( N, \{m^2\}, \mu ^2 \right) \!\equiv \! \int _0^1 \mathrm{d}\hat{\rho }\;\hat{\rho }^{N-1}\; \sigma _{ i j\rightarrow kl}\left( \hat{\rho },\{ m^2\}, \mu ^2 \right) . \end{aligned}$$

The variable $\hat{\rho }\equiv (m_k + m_l)^2/\hat{s}$ measures the closeness to the partonic production threshold and is related to the corresponding hadronic variable $\rho = \hat{\rho }x_i x_j$ in Eq. (3), where $x_i\ (x_j)$ is the usual longitudinal momentum fraction of the incoming proton carried out by the parton $i (j)$. The necessary inverse Mellin transform in Eq. (3) is performed along the contour $\mathrm{CT}$ according to the so-called “minimal prescription” [26]. The NLL-resummed cross section in Eq. (3) reads

5 $$\begin{aligned}&\tilde{\sigma }^\mathrm{(NLL)} _{ij\rightarrow kl}\left( N,\{m^2\},\mu ^2\right) = \sum _{I}\, \tilde{\sigma }^{(0)}_{ij\rightarrow kl,I}\left( N,\{m^2\},\mu ^2\right) \, \nonumber \\&\quad \times \, \Delta ^\mathrm{(NLL)}_i (N+1,Q^2,\mu ^2)\,\Delta ^\mathrm{(NLL)}_j (N+1,Q^2,\mu ^2)\, \nonumber \\&\quad \times \Delta ^\mathrm{(s, \mathrm {NLL})}_{ij\rightarrow kl,I}\left( N+1,Q^2,\mu ^2\right) , \end{aligned}$$

where the hard scale $Q^2$ is taken as $Q^2 = (m_k + m_l)^2$ and $\tilde{\sigma }^{(0)}_{ij \rightarrow kl, I}$ are the colour-decomposed leading-order cross sections in Mellin-moment space, with $I$ labelling the possible colour structures. The functions $\Delta ^\mathrm{(NLL)}_{i}$ and $\Delta ^\mathrm{(NLL)}_{j}$ sum the effects of the (soft-)collinear radiation from the incoming partons. They are process-independent and do not depend on the colour structures. These functions contain both the leading logarithmic as well as part of the sub-leading logarithmic behaviour. The expressions for $\Delta ^\mathrm{(NLL)}_{i}$ can be found in the literature [22]. In order to perform resummation at NLL accuracy, one also has to take into account soft-gluon contributions involving emissions from the final state, depending on the colour structures in which the final state SUSY particle pairs can be produced. They are summarised by the factor

6 $$\begin{aligned} \Delta _{I}^\mathrm{(s, \mathrm {NLL})}(N,Q^2,\mu ^2) \;=\; \exp \left[ \int _{\mu }^{Q/N}\frac{\mathrm{d}q}{q}\,\frac{\alpha _{\mathrm {s}}(q)}{\pi } \,D_{I} \,\right] . \end{aligned}$$

The one-loop coefficients $D_{I}$ follow from the threshold limit of the one-loop soft anomalous-dimension matrix and can be found in [22, 23].

The analytic results for the NLL part of the cross sections have been implemented into a numerical code. The results of this code, added to the NLO results obtained from Prospino2, correspond to the matched NLO + NLL cross sections. Their central values, the scale uncertainty and the 68 % C.L. PDF and $\alpha _{\mathrm {s}}$ uncertainties obtained using CTEQ6.6 [27] and MSTW2008 [28] PDFs have been tabulated for the squark and gluino production processes of interest in the range of input masses appropriate for the analyses.^1^ Together with a fast interpolation code, the tabulated values constitute the NLL-fast numerical package [25, 30].

In this paper we present results based on NLO + NLL calculations which can be obtained with the NLL-fast package. Results for squark and gluino production at next-to-next-to-leading logarithmic (NNLL) level in collisions at 8 TeV have been recently presented in [31–33] and a numerical code is in development. Additional NNLL results are available for selected processes such as stop–antistop [34] and gluino-gluino pair [35] production. For these processes, approximate next-to-next-to-leading order (NNLO) predictions including the dominant NNLO corrections coming from the resummed cross section at next-to-next-to-leading logarithmic (NNLL) level, also exist [36–38]. Moreover, a general formalism has been developed in the framework of effective field theories which allows for the resummation of soft and Coulomb gluons in the production of coloured sparticles [39, 40] and subsequently applied to squark and gluino production at NLL [40, 41] and NNLL accuracy [42]. The production of gluino bound states as well as bound-state effects in gluino pair and squark–gluino production has also been studied [43–46], with a recent study at NNLL accuracy [47] concentrating on the stoponium bound states. Finite width effects in the production of squark and gluino pairs have been investigated in [48]. Furthermore, electroweak corrections to the $\mathcal{O} (\alpha _{\mathrm {s}}^2)$ tree-level processes [49–55] and the electroweak Born production channels of $\mathcal{O} (\alpha \alpha _{\mathrm {s}})$ and $\mathcal{O} (\alpha ^2)$ [56, 57] are in general significant for the pair production of SU(2)-doublet squarks $\tilde{q}_L$ and at large invariant masses, but they are moderate for inclusive cross sections and will not be included in the results presented here.

## Treatment of cross sections and their associated uncertainties

The cross sections are taken at the next-to-leading order in the strong coupling constant, including the resummation of soft-gluon emission at the NLL level of accuracy, performed using the NLL-fast code. Currently, the code provides predictions for all squark and gluino production processes at $\sqrt{s}=7, 8$ and $13$ TeV. Additionally, results for stop (sbottom) pair production, gluino pair production with decoupled squarks and squark production with decoupled gluinos are available for $\sqrt{s}=13, 14, 33$ and 100 TeV [30]. For these particular cases, NLL-fast delivers cross sections for masses spanning $200$ GeV to $3, 3.5, 6.5, 15$ TeV for squark and gluino production and $100$ GeV to $2.5, 2.5, 5, 10$ TeV for direct stop or sbottom pair production at $\sqrt{s}=13, 14, 33, 100$ TeV, correspondingly. Further updates will appear soon [30]. Following the convention used in Prospino2, in the case of squarks, which can be more or less degenerate depending on a specific SUSY scenario, the input mass used is the result of averaging only the first- and second-generation squark masses. Further details on different scenarios considered to interpret the variety of experimental searches developed by the ATLAS and CMS collaborations are described in Sect. 3.1.

Scenarios have been investigated in which either the squark or gluino mass are set to some high scale, such that the corresponding sparticles cannot be produced at the LHC. Defining such a large mass scale is of course to some extend arbitrary and may have a non-negligible impact on the production of the SUSY particles residing at the TeV scale (e.g. squarks at high scales can still contribute to the gluino pair production process via a t-channel exchange). Thus, the calculation implemented in NLL-fast assumes that very heavy squarks or gluinos are completely decoupled and do not interfere with the production processes of the kinematically accessible particles.

The uncertainties due to the choice of the renormalisation and factorisation scales as well as the PDFs are obtained using the NLL-fast code. In order to combine all these predictions and obtain an overall uncertainty estimate, the PDF4LHC recommendations are followed as closely as possible, based on the availability of different calculations. Thus, an envelope of cross section predictions is defined using the 68 % C.L. ranges of the CTEQ6.6 [27] (including the $\alpha _{\mathrm {s}}$ uncertainty) and MSTW2008 [28] PDF sets, together with the variations of the scales. The nominal cross section is obtained using the midpoint of the envelope and the uncertainty assigned is half the full width of the envelope. If $\mathrm {PDF}_{\mathrm {CTEQ, up}}$($\mathrm {PDF}_{\mathrm {CTEQ, down}}$) and $\mathrm {SCA}_{\mathrm {CTEQ, up}}$($\mu _{\mathrm {CTEQ, down}}$) are the upward (downward) one sigma variations of the CTEQ6.6 PDF set, respectively, $\mathrm {PDF}_{\mathrm {MSTW, up}}$($\mathrm {PDF}_{\mathrm {MSTW, down}}$) and $\mu _{\mathrm {MSTW, up}}$($\mu _{\mathrm {MSTW, down}}$) are the corresponding variations for the MSTW2008PDF set and, finally, $\alpha _{\mathrm {S, up}}$($\alpha _{\mathrm {S, down}}$) is the corresponding up (down) one sigma uncertainty of the $\alpha _{\mathrm {s}}$coupling constant, the following quantities can be calculated:

7a $$\begin{aligned}&\mathrm {CTEQ}_{\mathrm {up}}= \sqrt{\mathrm {PDF}_{\mathrm {CTEQ, up}}^2 + \mathrm {SCA}_{\mathrm {CTEQ, up}}^2+\alpha _{\mathrm {S, up}}^2} \,,\end{aligned}$$

7b $$\begin{aligned}&\mathrm {CTEQ}_{\mathrm {down}}= \sqrt{\mathrm {PDF}_{\mathrm {CTEQ, down}}^2 + \mu _{\mathrm {CTEQ, down}}^2+\alpha _{\mathrm {S, down}}^2}\,, \end{aligned}$$

7c $$\begin{aligned}&\mathrm {MSTW}_{\mathrm {up}}= \sqrt{\mathrm {PDF}_{\mathrm {MSTW, up}}^2 + \mu _{\mathrm {MSTW, up}}^2} \,,\end{aligned}$$

7d $$\begin{aligned}&\mathrm {MSTW}_{\mathrm {down}}= \sqrt{\mathrm {PDF}_{\mathrm {MSTW, down}}^2 + \mu _{\mathrm {MSTW, down}}^2}\,. \end{aligned}$$

The corresponding upper and lower values of the envelope created by this set of numbers and the nominal predictions ($\mathrm {CTEQ}_{\mathrm {nom}}$ and $\mathrm {MSTW}_{\mathrm {nom}}$) are obtained by

8a $$\begin{aligned}&{U} \!=\! \mathrm {max} (\mathrm {CTEQ}_{\mathrm {nom}}\!+\! \mathrm {CTEQ}_{\mathrm {up}}, \mathrm {MSTW}_{\mathrm {nom}}\!+\! \mathrm {MSTW}_{\mathrm {up}}), \end{aligned}$$

8b $$\begin{aligned}&{L} \!=\!\mathrm {min} (\mathrm {CTEQ}_{\mathrm {nom}}\!-\! \mathrm {CTEQ}_{\mathrm {down}}, \mathrm {MSTW}_{\mathrm {nom}}\!-\! \mathrm {MSTW}_{\mathrm {down}}), \end{aligned}$$

and the final corresponding cross section ($\sigma$) and its symmetric uncertainty ($\Delta \sigma$) are taken to be:

9a $$\begin{aligned}&\sigma = ({U}+{L})/2 \,,\end{aligned}$$

9b $$\begin{aligned}&\Delta \sigma = ({U}-{L})/2\,. \end{aligned}$$

Full compliance with the PDF4LHC recommendations, with the inclusion of other PDF sets such as NNPDF [58], will be implemented in NLL-fast or its successor. We notice that, as discussed in Sect. 4, the additional contribution to the systematics coming from $\alpha _{\mathrm {s}}$ uncertainties is negligible.

### Special cases

Some SUSY models require special treatment in order to ensure that the NLO cross sections are correctly computed. Given the difficulty to provide a comprehensive summary of all situations that are being considered in the interpretation of the LHC data, we only discuss here few relevant cases, to exemplify the approach followed.

#### Simplified models

A variety of simplified models [59] are considered by the experiments. In some cases, the gluino, sbottom, and stops are decoupled from the rest of the supersymmetric spectrum.^2^ In this specific simplified model, only squark–antisquark production is allowed and this process is flavour-blind, if the masses are considered degenerate. Since the NLO + NLL calculations consider the sbottom as degenerate in mass with the squarks of the first and second generations, the overall cross section has to be rescaled by a factor of $4/5=0.8$.

In other cases where the gluino is not decoupled, squark–gluino and squark-squark productions are feasible, and can be used as provided by default. The only effect could come from a $b$-quark in the initial state, which, however, is strongly suppressed numerically [24]. Other types of simplified models decouple not only the third-generation squarks, but in addition all right-handed squarks. These scenarios primarily focus on squark decays via charginos or neutralinos. The squark mass is then calculated by averaging the non-decoupled squark masses and the final cross section is scaled by a factor of $(4/5)\cdot (1/2)=0.4$.

#### Treatment of third-generation squarks

Direct stop and sbottom production must be treated differently from the rest of squark families because, for instance, the $t$-channel gluino-exchange diagrams are suppressed.

In computations of squark production processes involving sbottoms, masses of sbottoms can be considered either degenerate with the rest of other squark flavours, as done in NLL-Fast, or non-degenerate. In scenarios in which the production of different squark flavours are present, the squark-pair production cross section is rescaled down to subtract the sbottom contribution and the corresponding process is computed separately.

At leading order the corresponding partonic cross sections for the production of third-generation squarks depend only on their masses, and the results for sbottom and stop of the same mass are therefore equal. At NLO in SUSY-QCD, additional SUSY parameters like squark and gluino masses or the stop/sbottom mixing angle enter. Their numerical impact, however, is very small [12, 24]. A further difference between stop and sbottom pair production arises from the $b\bar{b} \rightarrow \tilde{b}\tilde{b}^*$ channel, where the initial-state bottom quarks do allow a $t$-channel gluino-exchange graph that gives rise to extra contributions. However, as has been demonstrated in Ref. [24] their numerical impact on the hadronic cross sections is negligible. Thus, for all practical purposes, the LO and higher-order cross section predictions obtained for stop pair production apply also to sbottom pair production if the input parameters, i.e. masses and mixing angles, are modified accordingly.

## Squark and gluino production at the LHC

**Fig. 1:**
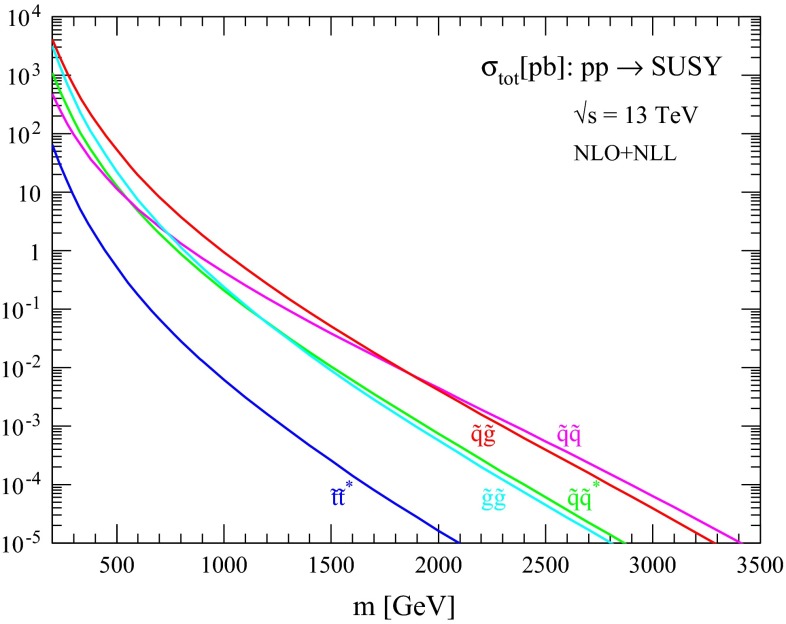
NLO + NLL production cross sections for the case of equal degenerate squark and gluino masses as a function of mass at $\sqrt{s}=13$ TeV

**Fig. 2:**
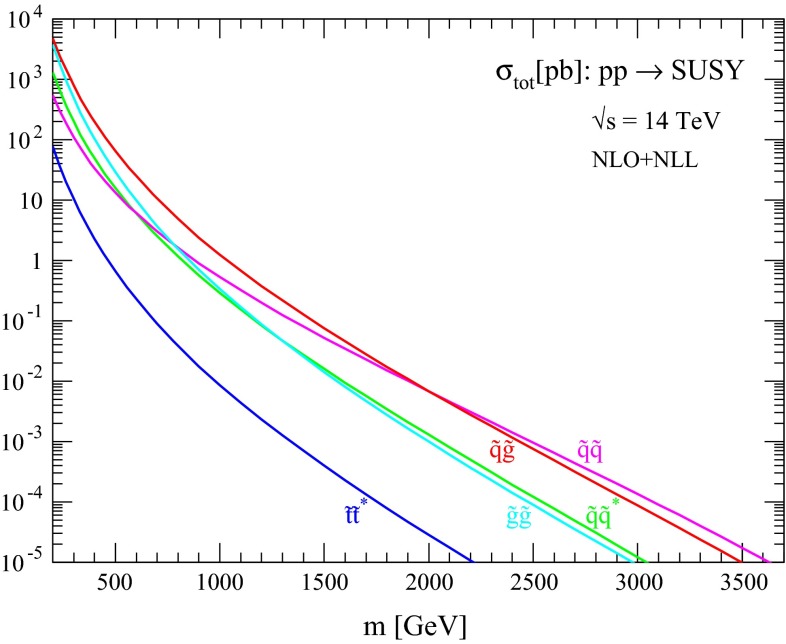
NLO + NLL production cross sections for the case of equal degenerate squark and gluino masses as a function of mass at $\sqrt{s}=14$ TeV

**Fig. 3:**
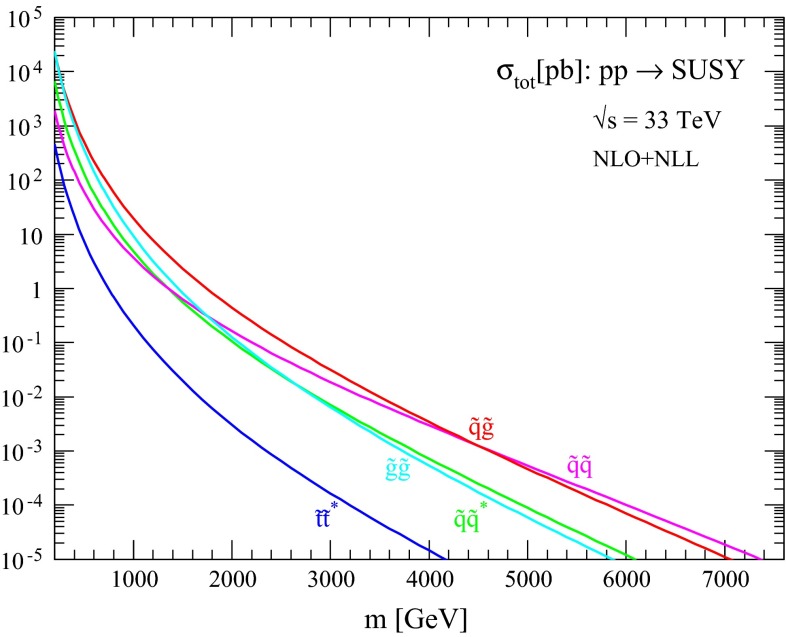
NLO + NLL production cross sections for the case of equal degenerate squark and gluino masses as a function of mass at $\sqrt{s}=33$ TeV

**Fig. 4:**
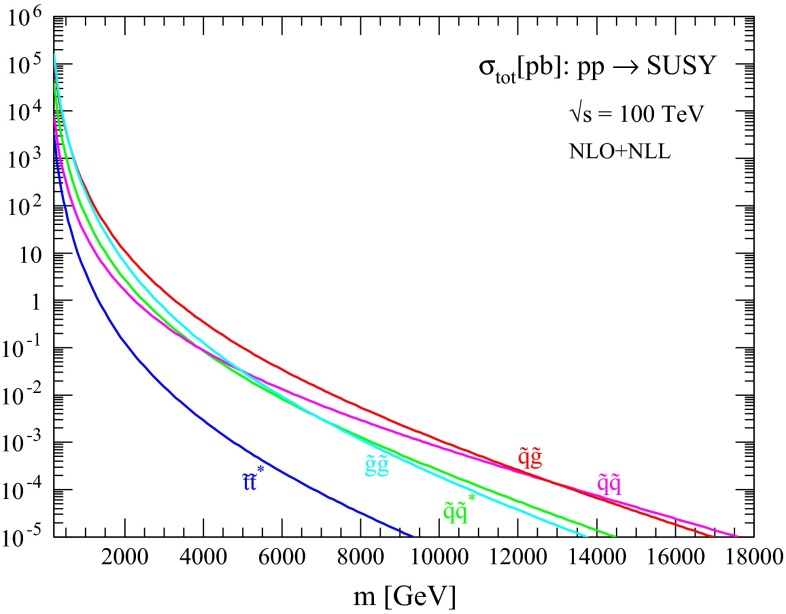
NLO + NLL production cross sections for the case of equal degenerate squark and gluino masses as a function of mass at $\sqrt{s}=100$ TeV

The production cross sections and associated uncertainties resulting from the procedure described in the previous section are discussed here for different processes of interest. First, in Figs. 1, 2, 3 and 4 we show the NLO + NLL central predictions for the various squark and gluino production processes for the case of equal squark and gluino masses at collider energies $\sqrt{s}=13, 14, 33$ and $100$ TeV, respectively. Assuming a squark and gluino mass near 2 TeV, we predict inclusive SUSY cross sections of the order $10^{-2}$, 1 and 20 pb at $\sqrt{s}=13 (14), 33$ and $100$ TeV, respectively. The relative size of the various production channels depends on the relative size of squark/gluino masses and collider energy. For small SUSY masses and/or large collider energies, gluino cross sections are dominant, while for large SUSY masses and/or low collider energies the valence quark distributions favour squark–gluino associate and squark-pair production.

We furthermore discuss three distinct special cases in some detail: gluino pair production with decoupled squarks, squark–antisquark pair production with gluino decoupled and stop/sbottom pair production. The results shown here are mainly illustrative: tables with cross sections and systematic uncertainties obtained in other scenarios are collected at the SUSY cross section working group web page [4].

### Gluino pair production

**Fig. 5:**
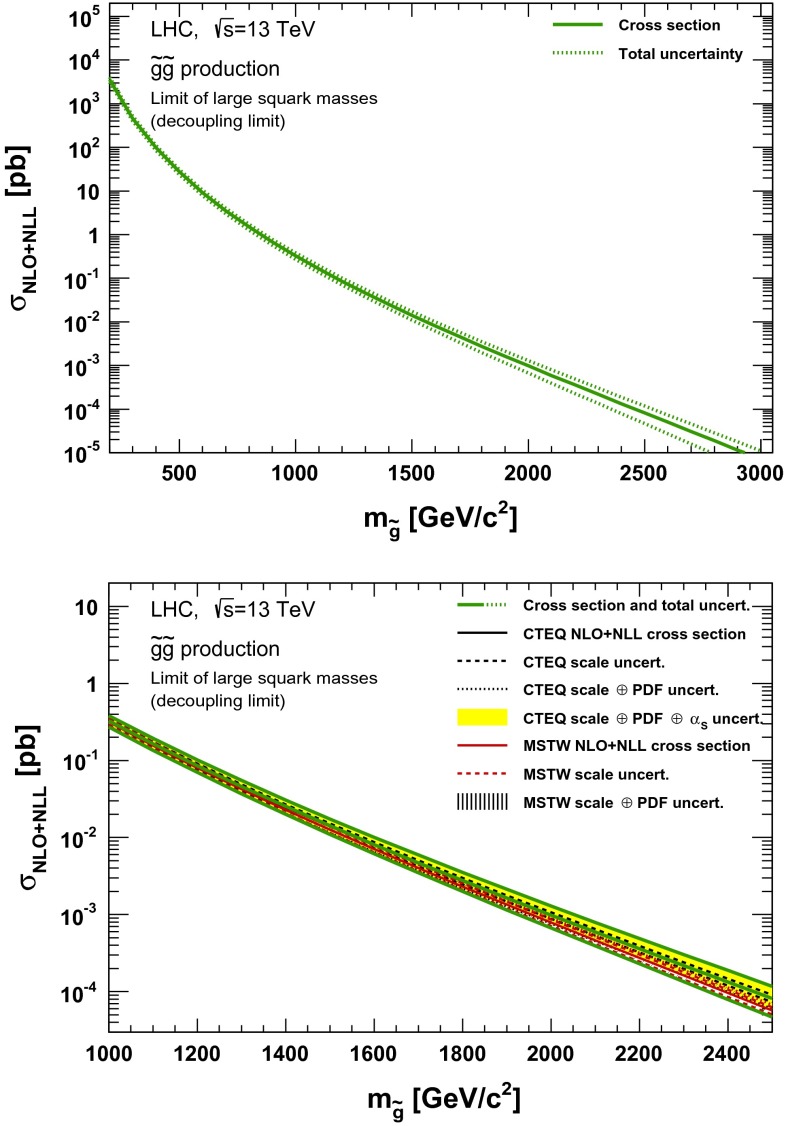
NLO + NLL gluino pair production cross section with squarks decoupled as a function of mass at $\sqrt{s}=13$ TeV in the wider (*upper plot*) and narrower (*lower plot*) mass range. The different styled *black* (*red*) *lines* correspond to the cross section and scale uncertainties predicted using the CTEQ6.6(MSTW2008) PDF set. The *yellow* (*dashed black*) *band* corresponds to the total CTEQ6.6(MSTW2008) uncertainty, as described in the text. The *green lines* show the final cross section and its total uncertainty

**Fig. 6:**
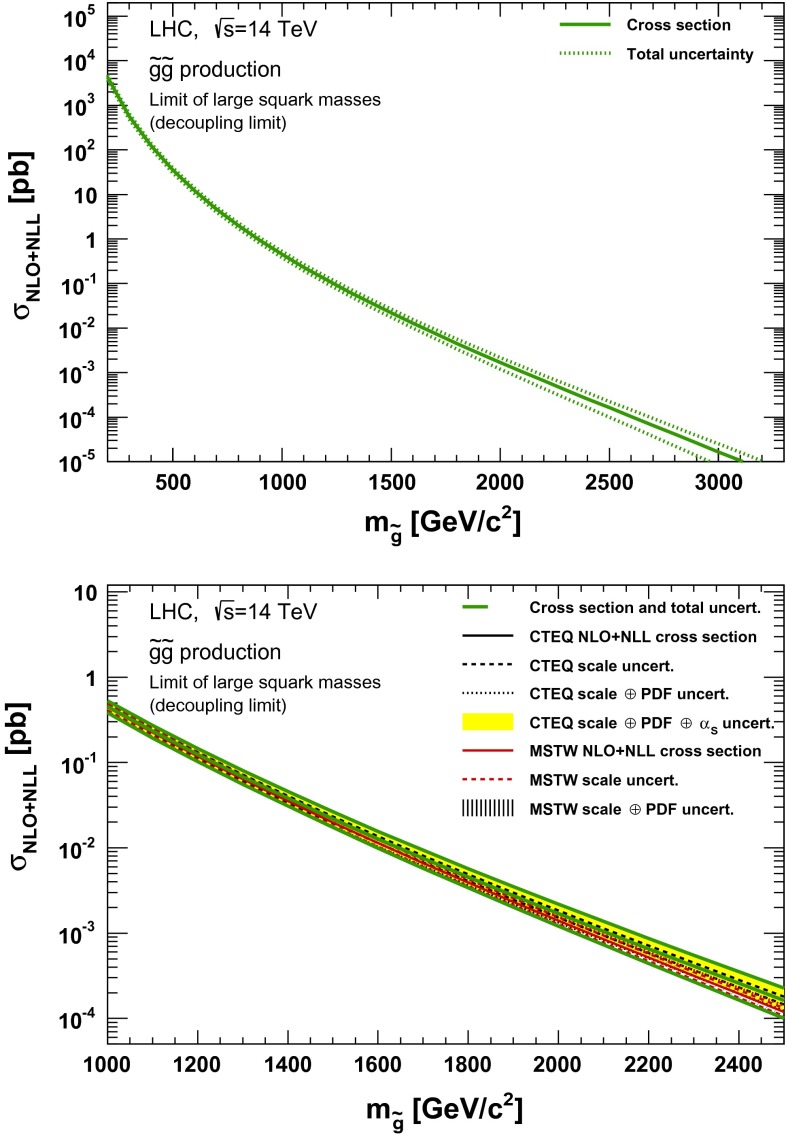
NLO + NLL gluino pair production cross section with squarks decoupled as a function of mass at $\sqrt{s}=14$ TeV in the wider (*upper plot*) and narrower (*lower plot*) mass range. The different styled *black* (*red*) *lines* correspond to the cross section and scale uncertainties predicted using the CTEQ6.6(MSTW2008) PDF set. The *yellow* (*dashed black*) *band* corresponds to the total CTEQ6.6(MSTW2008) uncertainty, as described in the text. The *green lines* show the final cross section and its total uncertainty

**Fig. 7:**
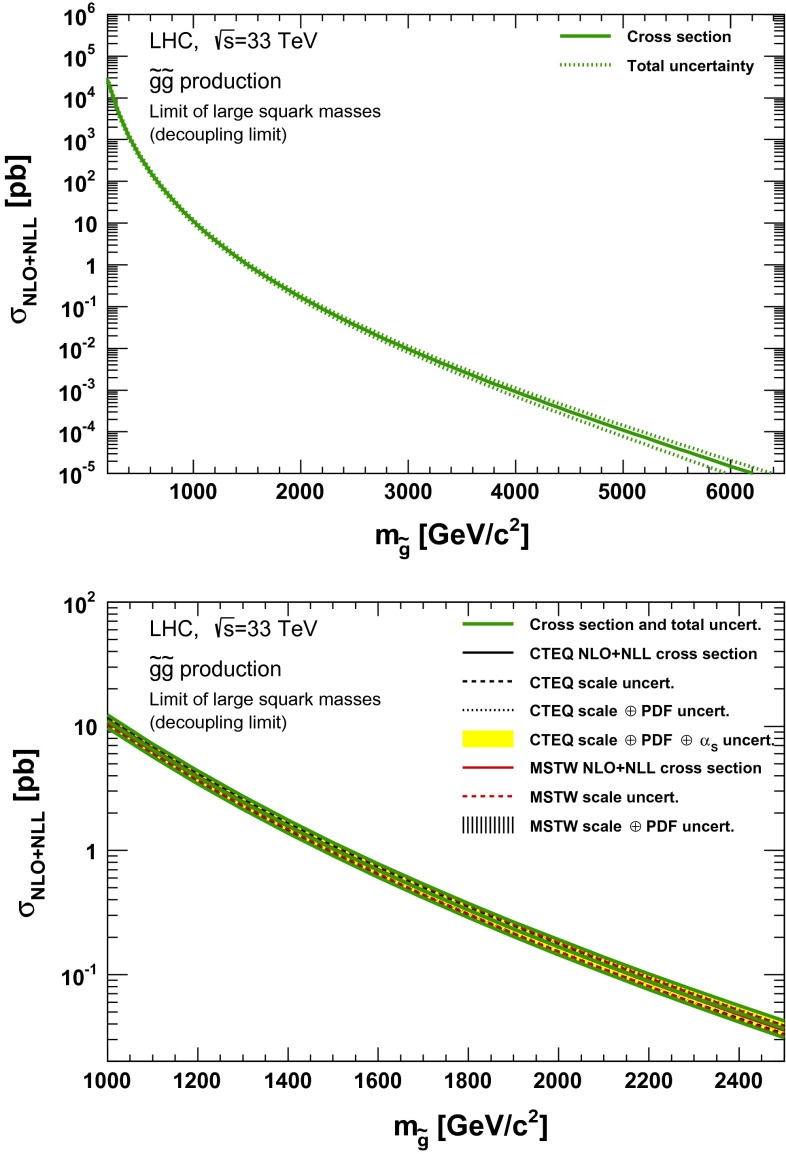
NLO + NLL gluino pair production cross section with squarks decoupled as a function of mass at $\sqrt{s}=33$ TeV in the wider (*upper plot*) and narrower (*lower plot*) mass range. The different styled *black* (*red*) *lines* correspond to the cross section and scale uncertainties predicted using the CTEQ6.6(MSTW2008) PDF set. The *yellow* (*dashed black*) *band* corresponds to the total CTEQ6.6(MSTW2008) uncertainty, as described in the text. The *green lines* show the final cross section and its total uncertainty

**Fig. 8:**
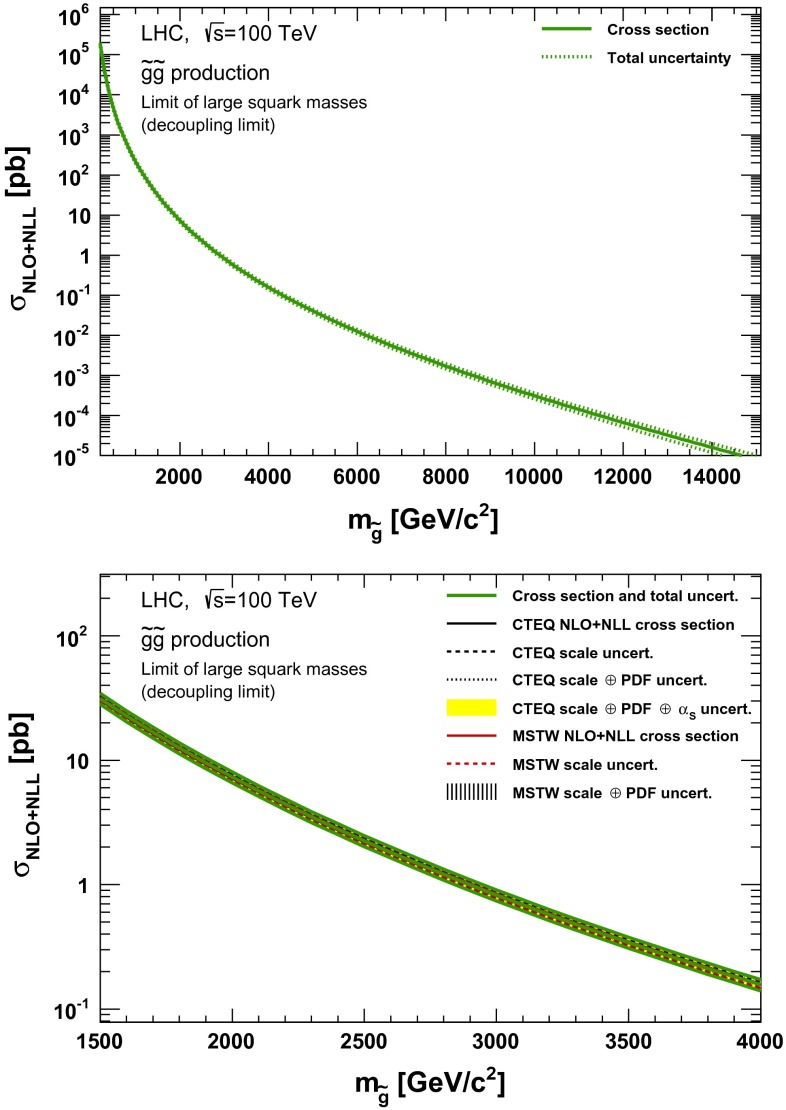
NLO + NLL gluino pair production cross section with squarks decoupled as a function of mass at $\sqrt{s}=100$ TeV in the wider (*upper plot*) and narrower (*lower plot*) mass range. The different styled *black* (*red*) *lines* correspond to the cross section and scale uncertainties predicted using the CTEQ6.6(MSTW2008) PDF set. The *yellow* (*dashed black*) *band* corresponds to the total CTEQ6.6(MSTW2008) uncertainty, as described in the text. The *green lines* show the final cross section and its total uncertainty

The gluino pair production cross section in a model where the squarks are decoupled is shown in Fig. 5 for $\sqrt{s}=13$ TeV, Fig. 6 for $\sqrt{s}=14$ TeV, in Fig. 7 for $\sqrt{s}=33$ TeV and in Fig. 8 for $\sqrt{s}=100$ TeV. The gluino mass spans the range from 0.2 to 3 TeV in the upper plot of Fig. 5. The close up of the mass range from 1 to 2.5 TeV for $\sqrt{s}=13$ TeV is provided in the lower plot of Fig. 5. Results for $\sqrt{s}=14$ TeV in the mass ranges 0.2–3.3 and 1–2.5 TeV are shown in Fig. 6 and results for $\sqrt{s}=33$ TeV in the mass ranges 0.2–6.5 TeV and 1–2.5 TeV are presented in Fig. 7, respectively. Similarly, Fig. 8 shows results for $\sqrt{s}=100$ TeV in the mass ranges 0.2–15 and 1.5–4 TeV. In the lower figures, the black (red) line corresponds to the NLO + NLL nominal cross section and renormalisation and factorisation scale uncertainties obtained using the CTEQ6.6 (MSTW2008) PDF set. The solid yellow (dashed black) band corresponds to the total uncertainty of the cross section using CTEQ6.6 (MSTW2008), as derived from Eq. (7a). Finally, the green lines in the upper and lower plots delimit the envelope and the central value. They correspond to the central nominal value together with the total uncertainties.

### Squark–antisquark production

**Fig. 9:**
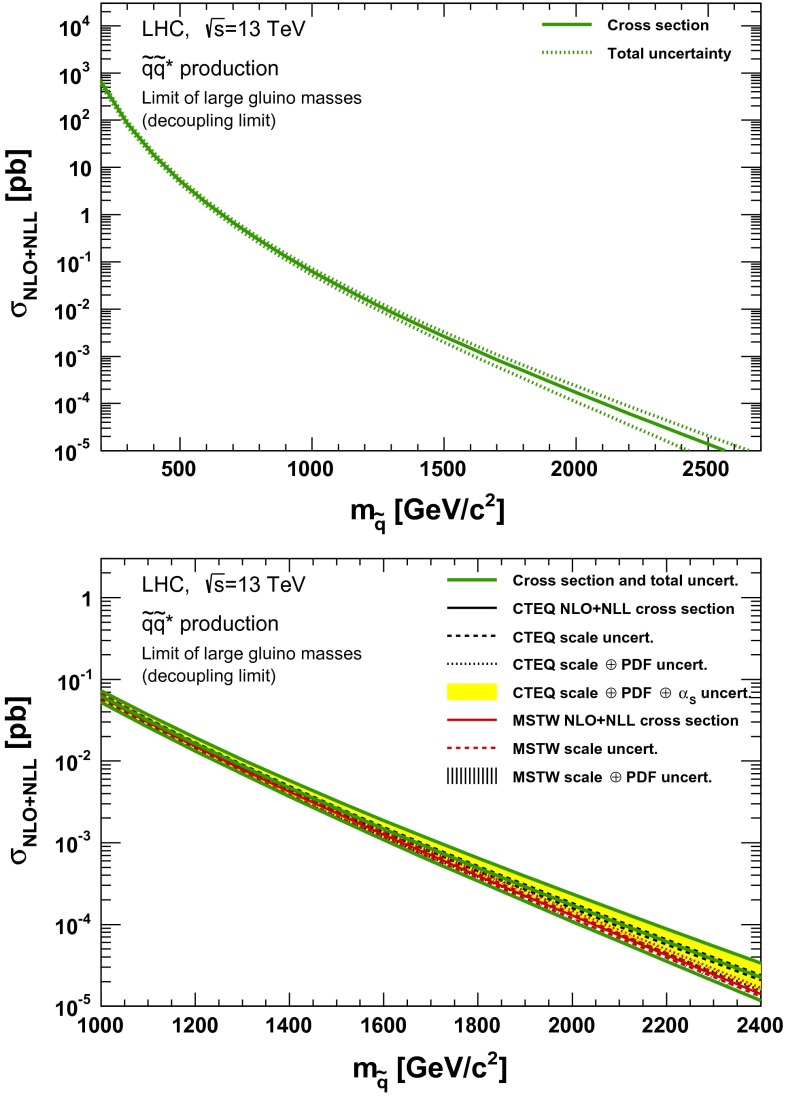
NLO + NLL squark–antisquark pair production cross section with squarks decoupled as a function of mass at $\sqrt{s}=13$ TeV in the wider (*upper plot*) and narrower (*lower plot*) mass range. The different styled *black* (*red*) *lines* correspond to the cross section and scale uncertainties predicted using the CTEQ6.6(MSTW2008) PDF set. The *yellow* (*dashed black*) *band* corresponds to the total CTEQ6.6(MSTW2008) uncertainty, as described in the text. The *green lines* show the final cross section and its total uncertainty

**Fig. 10:**
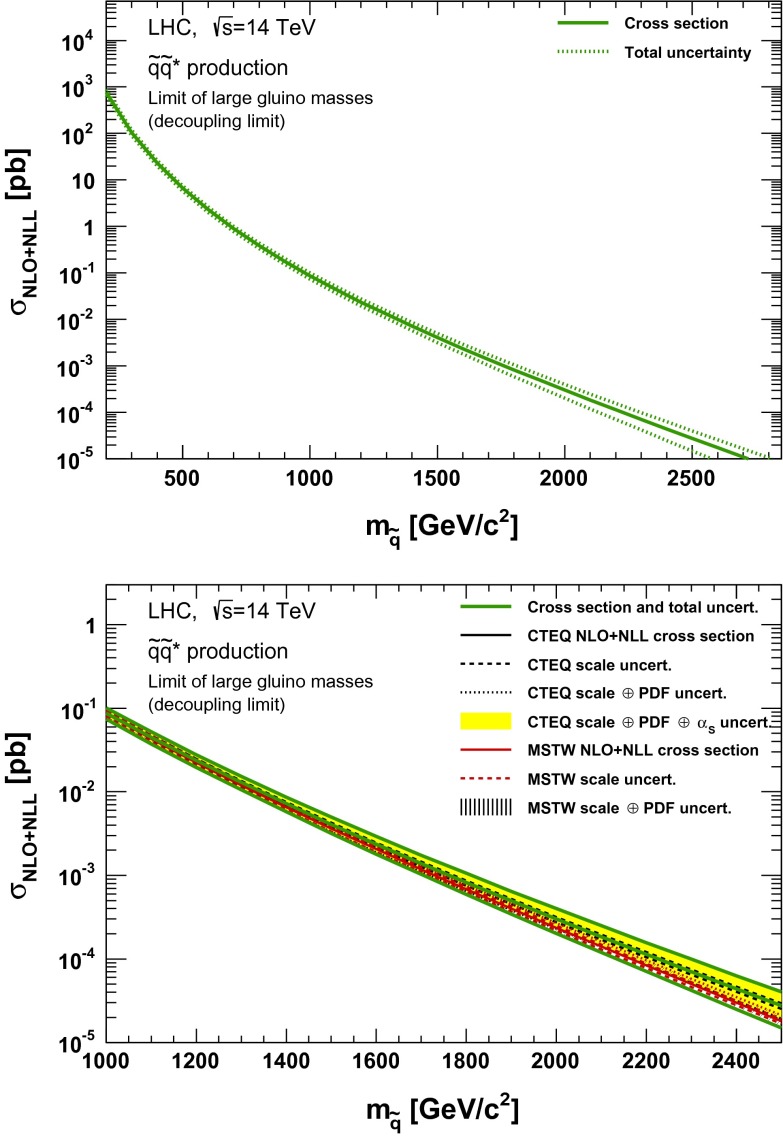
NLO + NLL squark–antisquark pair production cross section with squarks decoupled as a function of mass at $\sqrt{s}=14$ TeV in the wider (*upper plot*) and narrower (*lower plot*) mass range. The different styled *black* (*red*) *lines* correspond to the cross section and scale uncertainties predicted using the CTEQ6.6(MSTW2008) PDF set. The *yellow* (*dashed black*) *band* corresponds to the total CTEQ6.6(MSTW2008) uncertainty, as described in the text. The *green lines* show the final cross section and its total uncertainty

**Fig. 11:**
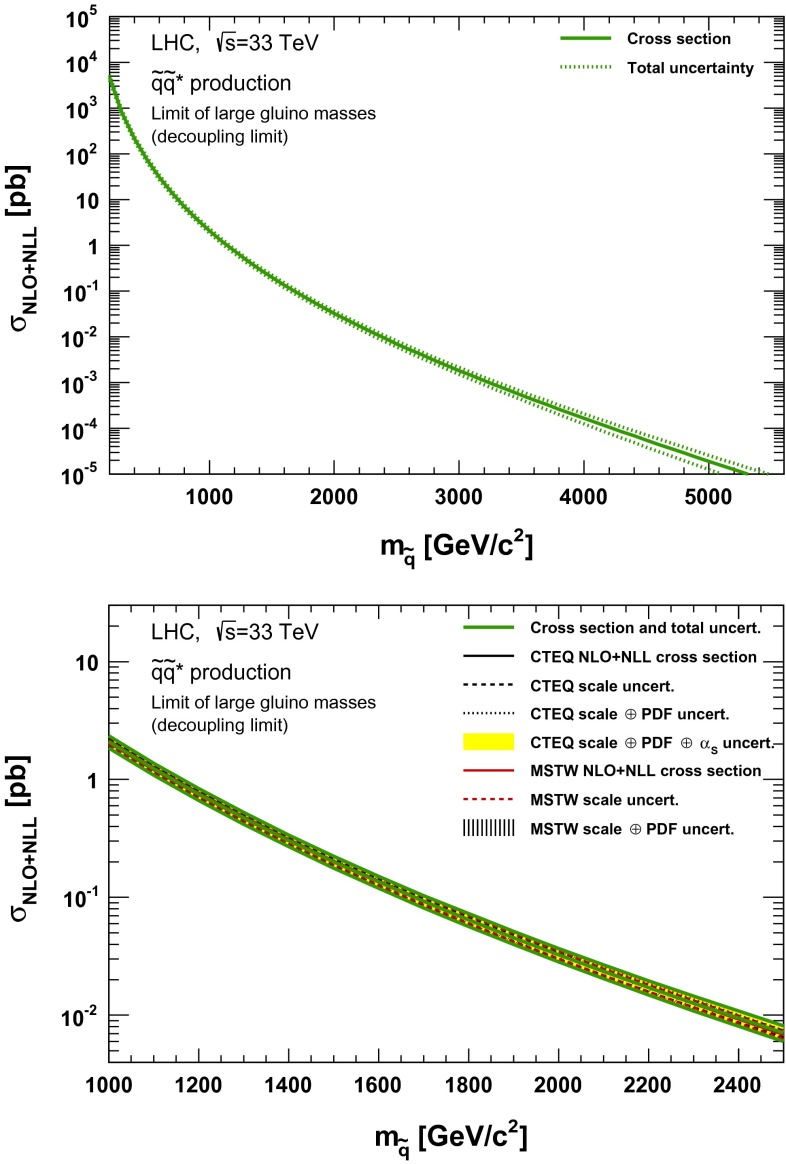
NLO + NLL squark–antisquark pair production cross section with squarks decoupled as a function of mass at $\sqrt{s}=33$ TeV in the wider (*upper plot*) and narrower (*lower plot*) mass range. The different styled *black* (*red*) *lines* correspond to the cross section and scale uncertainties predicted using the CTEQ6.6(MSTW2008) PDF set. The *yellow* (*dashed black*) *band* corresponds to the total CTEQ6.6(MSTW2008) uncertainty, as described in the text. The *green lines* show the final cross section and its total uncertainty

In order to show the evolution of the squark–antisquark production cross section as a function of the squark mass, a scenario has been chosen in which the gluino is decoupled. The results are shown in Figs. 9, 10, 11 and in  12, using the same convention for the display of the various contributions as in the gluino pair production case.

**Fig. 12:**
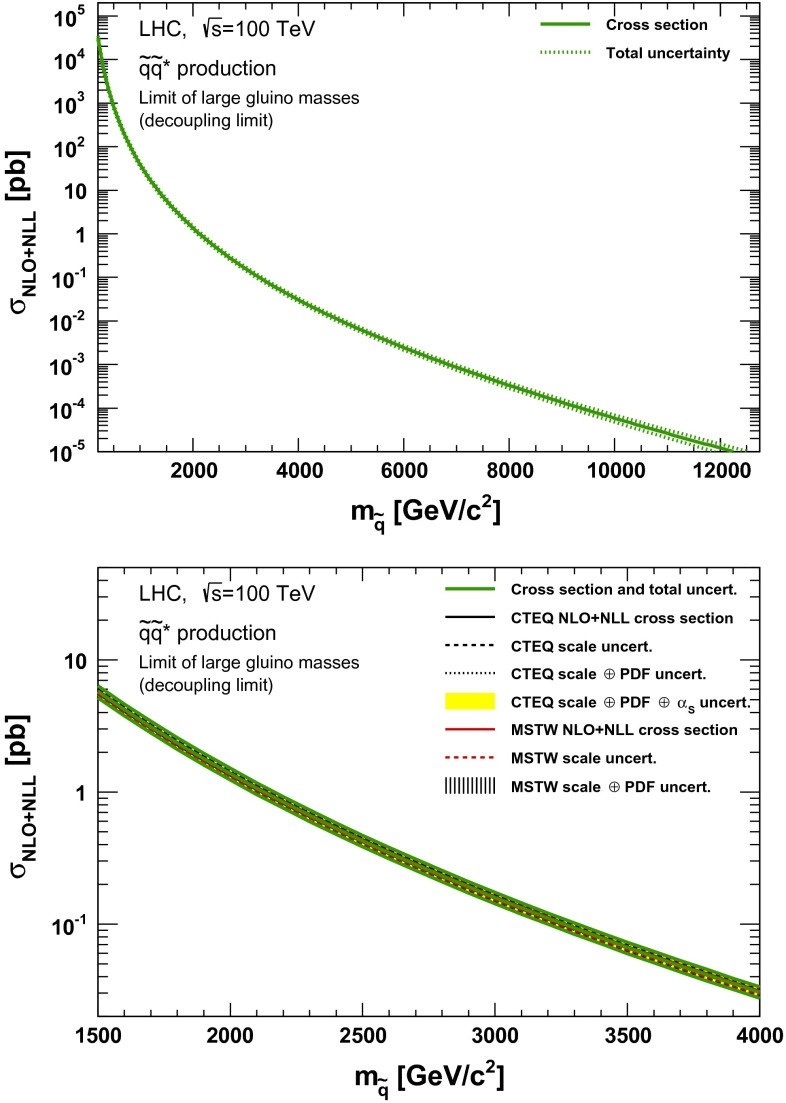
NLO + NLL squark–antisquark pair production cross section with squarks decoupled as a function of mass at $\sqrt{s}=100$ TeV in the wider (*upper plot*) and narrower (*lower plot*) mass range. The different styled *black* (*red*) *lines* correspond to the cross section and scale uncertainties predicted using the CTEQ6.6(MSTW2008) PDF set. The *yellow* (*dashed black*) *band* corresponds to the total CTEQ6.6(MSTW2008) uncertainty, as described in the text. The *green lines* show the final cross section and its total uncertainty

### Direct stop and sbottom pair production

**Fig. 13:**
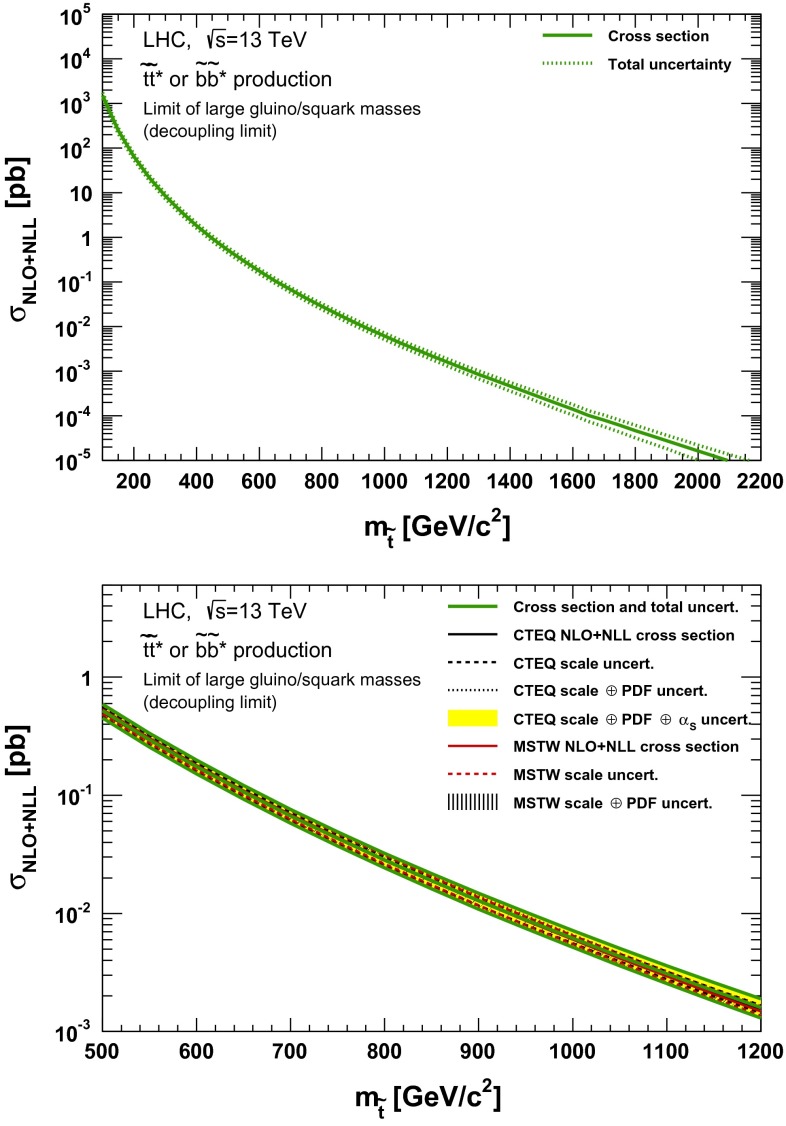
NLO + NLL stop–antistop production cross section as a function of mass at $\sqrt{s}=13$ TeV in the wider (*upper plot*) and narrower (*lower plot*) mass range. The different styled *black* (*red*) *lines* correspond to the cross section and scale uncertainties predicted using the CTEQ6.6(MSTW2008) PDF set. The *yellow* (*dashed black*) *band* corresponds to the total CTEQ6.6(MSTW2008) uncertainty, as described in the text. The *green lines* show the final cross section and its total uncertainty

**Fig. 14:**
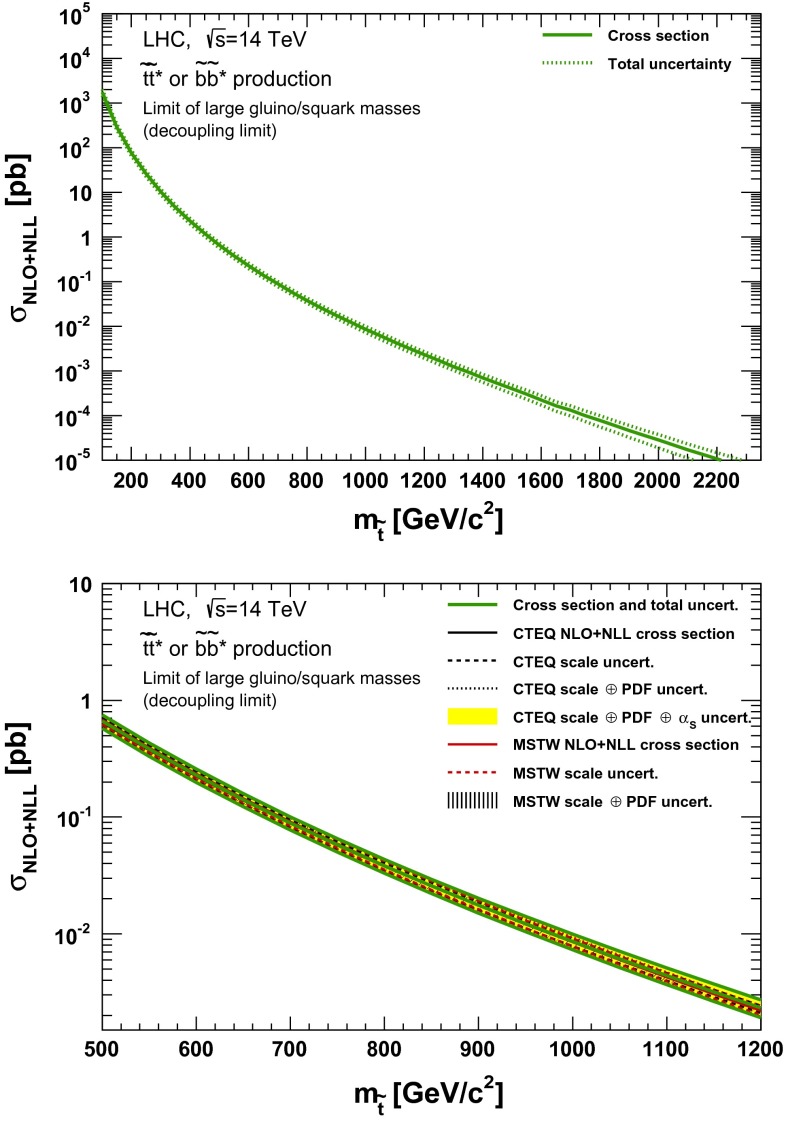
NLO + NLL stop–antistop production cross section as a function of mass at $\sqrt{s}=14$ TeV in the wider (*upper plot*) and narrower (*lower plot*) mass range. The different styled *black* (*red*) *lines* correspond to the cross section and scale uncertainties predicted using the CTEQ6.6 (MSTW2008) PDF set. The *yellow* (*dashed black*) *band* corresponds to the total CTEQ6.6 (MSTW2008) uncertainty, as described in the text. The *green lines* show the final cross section and its total uncertainty

**Fig. 15:**
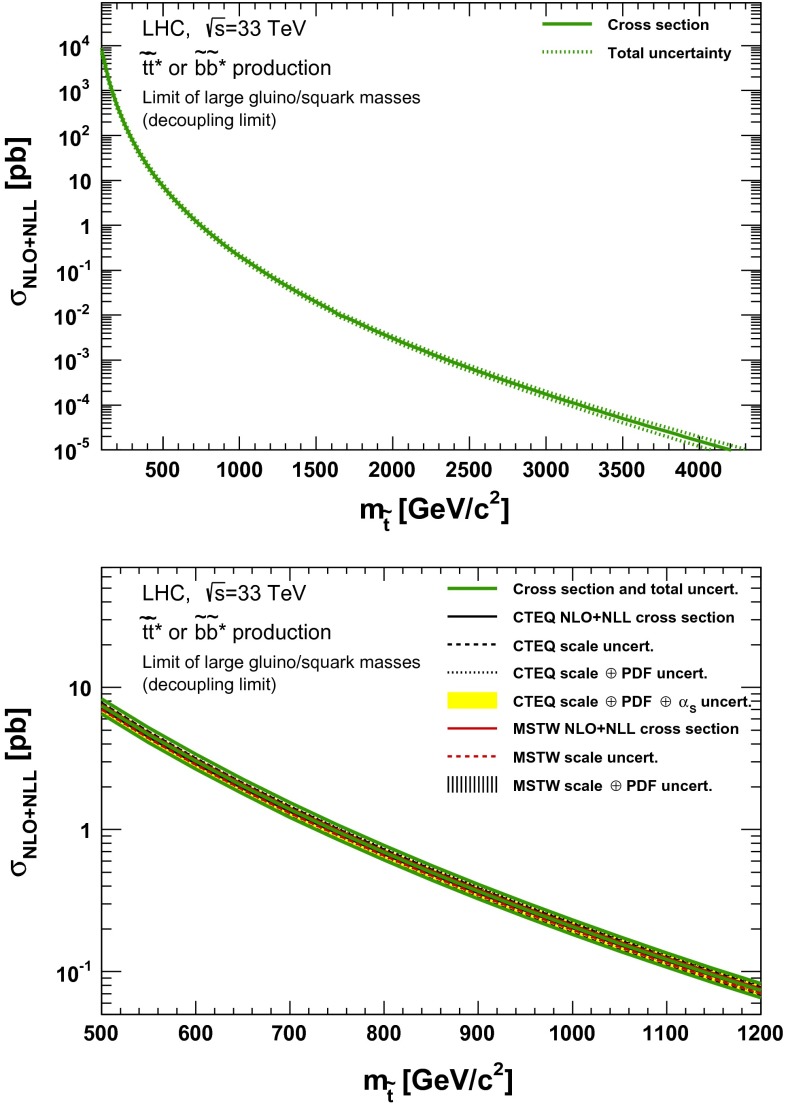
NLO + NLL stop–antistop production cross section as a function of mass at $\sqrt{s}=33$ TeV in the wider (*upper plot*) and narrower (*lower plot*) mass range. The different styled *black* (*red*) *lines* correspond to the cross section and scale uncertainties predicted using the CTEQ6.6(MSTW2008) PDF set. The *yellow* (*dashed black*) *band* corresponds to the total CTEQ6.6(MSTW2008) uncertainty, as described in the text. The *green lines* show the final cross section and its total uncertainty

**Fig. 16:**
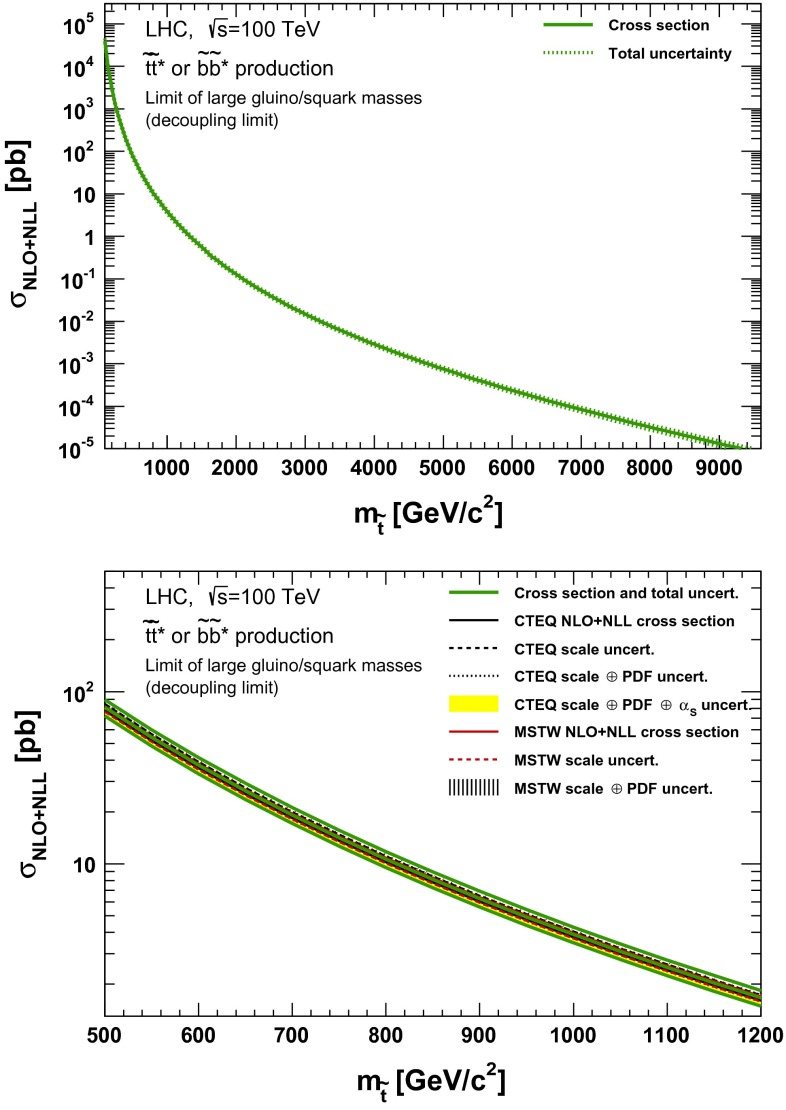
NLO + NLL stop–antistop production cross section as a function of mass at $\sqrt{s}=100$ TeV in the wider (*upper plot*) and narrower (*lower plot*) mass range. The different styled *black* (*red*) *lines* correspond to the cross section and scale uncertainties predicted using the CTEQ6.6(MSTW2008) PDF set. The *yellow* (*dashed black*) *band* corresponds to the total CTEQ6.6(MSTW2008) uncertainty, as described in the text. The *green lines* show the final cross section and its total uncertainty

The production cross section as a function of the stop mass for a model in which only the lightest stop is reachable is shown in Figs. 13, 14, 15 and 16. It should be noted that these cross sections are approximately the same as those of a model in which only the lightest sbottom is accessible, assuming the rest of the coloured SUSY spectrum decoupled.

## Summary and future prospects

We have presented reference cross sections for the production of squarks and gluinos at the upcoming LHC runs with $\sqrt{s}=13$ and $14$, and at future $pp$ colliders operating at $\sqrt{s}=33$ and $100$ TeV. The theoretical predictions are based on resummed results at the next-to-leading logarithmic (NLL) accuracy matched to next-to-leading order (NLO) predictions.^3^ We provide an estimate of the theoretical uncertainty following the prescriptions established in [3], and used by the ATLAS and CMS collaborations in the interpretation of their measurements for $\sqrt{s}=7$ and $8$ TeV. The theoretical systematic uncertainties are larger for higher sparticle masses, and they are typically dominated by the PDF uncertainties. These have a significant impact when assessing the experimental constraints or the sensitivity to a given SUSY model. Cross sections are evaluated using the CTEQ6.6 and MSTW2008 PDFs. The large-x behaviour of these PDF sets is determined in terms of few parameters, whose values are fixed in the region with experimental constraints. For the production of high-mass SUSY particles, these functional forms are extrapolated beyond the constraints provided by data.

Differences between PDF sets will be reduced as more and more experimental measurements become available, in particular with the results of the LHC Run II, as well as by improving the fitting methodology and the theoretical calculations.

In anticipation of this improved accuracy expected in future PDF determinations, the central values presented for the first time in this paper at NLL accuracy can serve as an estimate of high mass SUSY coloured production for current and future colliders. Detailed numbers and tables for a broad class of SUSY models and parameters are collected at the SUSY cross section working group web page [4].

## Footnotes

1 Note that we use NLO PDFs with the NLO + NLL matched cross section calculation. The reduction of the factorisation scale dependence observed in the NLO + NLL predictions is a result of a better compensation between the scale dependence of the NLO evolution of the PDF and the short distance cross section, and does not depend on whether PDFs are fitted using NLO or NLO + NLL theory; see for example Ref. [29]. In general, one can apply NLL threshold resummation with NLO PDFs to processes like heavy SUSY particle production for which the summation of logarithms is more important than for the input data to the NLO fits. However, it would be interesting to systematically study the difference between NLO and NLO + NLL input to global PDF determinations for SUSY particle production at the LHC.

2 While this scenario is possible if we only consider the TeV scale, it bears some challenges at higher energy scales. Any kind of renormalisation scale evolution will generate squark masses at the scale of the gluino mass, but not vice versa. Thus, any discovery of light squarks associated with heavy gluinos would point to a non-standard underlying model [60]. In spite of all theory prejudice it is clearly adequate that these regions be experimentally explored.

3 This paper should be cited along with the original papers, i.e. Refs. [11, 21–23, 25] for inclusive squark/gluino production, Refs. [12, 24, 25] for stop or sbottom direct production.
